# Bronchial mucosal inflammation and illness severity in response to experimental rhinovirus infection in COPD

**DOI:** 10.1016/j.jaci.2020.03.021

**Published:** 2020-10

**Authors:** Jie Zhu, Patrick Mallia, Joseph Footitt, Yusheng Qiu, Simon D. Message, Tatiana Kebadze, Julia Aniscenko, Peter J. Barnes, Ian M. Adcock, Onn M. Kon, Malcolm Johnson, Marco Contoli, Luminita A. Stanciu, Alberto Papi, Peter K. Jeffery, Sebastian L. Johnston

**Affiliations:** a National Heart and Lung Institute, Imperial College, London, United Kingdom; b Imperial College Healthcare NHS Trust, London, United Kingdom; c GlaxoSmithKline, Uxbridge, Middlesex, United Kingdom; d Research Centre on Asthma and COPD, University of Ferrara, Ferrara, Italy

**Keywords:** Rhinovirus infection, eosinophils, inflammation, chronic obstructive pulmonary disease exacerbation, COPD, Chronic obstructive pulmonary disease, Epi, Epithelial, RV, Rhinovirus, Sub, Subepithelial

## Abstract

**Background:**

Respiratory viral infection causes chronic obstructive pulmonary disease (COPD) exacerbations. We previously reported increased bronchial mucosa eosinophil and neutrophil inflammation in patients with COPD experiencing naturally occurring exacerbations. But it is unclear whether virus per se induces bronchial mucosal inflammation, nor whether this relates to exacerbation severity.

**Objectives:**

We sought to determine the extent and nature of bronchial mucosal inflammation following experimental rhinovirus (RV)-16–induced COPD exacerbations and its relationship to disease severity.

**Methods:**

Bronchial mucosal inflammatory cell phenotypes were determined at preinfection baseline and following experimental RV infection in 17 Global Initiative for Chronic Obstructive Lung Disease stage II subjects with COPD and as controls 20 smokers and 11 nonsmokers with normal lung function. No subject had a history of asthma/allergic rhinitis: all had negative results for aeroallergen skin prick tests.

**Results:**

RV infection increased the numbers of bronchial mucosal eosinophils and neutrophils only in COPD and CD8^+^ T lymphocytes in patients with COPD and nonsmokers. Monocytes/macrophages, CD4^+^ T lymphocytes, and CD20^+^ B lymphocytes were increased in all subjects. At baseline, compared with nonsmokers, subjects with COPD and smokers had increased numbers of bronchial mucosal monocytes/macrophages and CD8^+^ T lymphocytes but fewer numbers of CD4^+^ T lymphocytes and CD20^+^ B lymphocytes. The virus-induced inflammatory cells in patients with COPD were positively associated with virus load, illness severity, and reductions in lung function.

**Conclusions:**

Experimental RV infection induces bronchial mucosal eosinophilia and neutrophilia only in patients with COPD and monocytes/macrophages and lymphocytes in both patients with COPD and control subjects. The virus-induced inflammatory cell phenotypes observed in COPD positively related to virus load and illness severity. Antiviral/anti-inflammatory therapies could attenuate bronchial inflammation and ameliorate virus-induced COPD exacerbations.

Exacerbations in chronic obstructive pulmonary disease (COPD) are a major cause of morbidity and mortality.^1^ Respiratory viral infections are the major cause of acute exacerbations,^2^ with human rhinoviruses (RVs) the most common viruses detected.^3^ Our own previously reported studies have shown that experimental RV infection in subjects with COPD induces lower respiratory tract symptoms, airflow obstruction, and systemic and airway inflammation that are greater and more prolonged compared with smoking control subjects without airway obstruction, indicating a causal relationship between RV infection and COPD exacerbations.^4^

COPD in its stable phase is characterized by airway inflammation that is central to the pathogenesis of the disease,^5^ with increased numbers of airway mucosal monocytes/macrophages, CD4^+^ and CD8^+^ T and B lymphocytes, and neutrophils that are associated with the severity of airflow limitation.^10^, ^6^, ^7^, ^8^, ^9^ Neutrophilic inflammation has been a classical hallmark of both stable COPD^8^^,^^11^ and naturally occurring COPD exacerbations.^12^^,^^13^ Eosinophilic inflammation, although usually considered a characteristic feature of asthma, is also present in a subset of patients with COPD both when stable and during exacerbations.^13^, ^14^, ^15^ Increased numbers of mucosal eosinophils have been detected in bronchial biopsies from subjects with chronic bronchitis and subjects with COPD experiencing naturally occurring exacerbations.^16^, ^17^, ^18^ However, the role of eosinophils in COPD exacerbations, particularly in respiratory virus–induced exacerbations remains unclear. It is unknown whether virus infection per se can cause mucosal eosinophilia and neutrophilia during COPD exacerbations. Also, there have been a number of confounding factors in some of the aforementioned studies, such as inclusion of mechanically ventilated patients who had received oral corticosteroids before sampling,^18^ and use of different patient groups for comparison of stable versus exacerbated states.^16^, ^17^, ^18^

We have developed an experimental model of a COPD exacerbation using human RV-16 infection in nonintubated, treatment-naive patients with COPD. As part of 2 completed studies using this model,^4^^,^^19^ bronchial biopsies were collected from patients with COPD, smokers without COPD,^4^^,^^19^ and nonsmokers^19^ at baseline before infection and on day 7 during the acute infection. These samples provide a unique opportunity to explore the bronchial mucosal inflammatory response and its physiological and clinical significance in virus-induced experimental COPD exacerbations, and to investigate whether these responses differ between patients with and without COPD.

We hypothesized that RV infection alone recruits inflammatory cells into the bronchial mucosa and that the nature of the inflammatory response and its associated severity of clinical symptoms and airflow obstruction in subjects with COPD is distinct from that seen in subjects without COPD.

## Methods

### Participants

Table I presents demographic data at baseline and after infection in this study (ie, those successfully infected and having adequate bronchial biopsy material for analysis), namely, 17 smokers with Global Initiative for Chronic Obstructive Lung Disease stage II COPD (FEV_1_ 50%-79% predicted normal value, FEV_1_/forced vital capacity <70%, and β-agonist reversibility <12%), 20 smokers with normal lung function (FEV_1_ ≥80% predicted; FEV_1_/forced vital capacity >70%), and 11 healthy nonsmokers. The inclusion/exclusion criteria are provided in Table E1 in this article’s Online Repository at www.jacionline.org. All subjects were nonatopic as defined by negative reactions to a 6-grass pollen mix on skin prick tests, and none had any history of asthma or allergic rhinitis. No subject had symptoms of respiratory tract infection within the previous 8 weeks. Patients with COPD had no exacerbation and were treatment-naive in the previous 3 months. No subject used corticosteroids (either inhaled or systemic) or antibiotics to treat the exacerbations after experimental RV infection. The only medication allowed was increased use of short-acting β_2_-agonists. All subjects gave informed written consent, and the study protocols were approved by St Mary’s NHS Trust Research Ethics Committee (study nos. 00/BA/459E and 07/H0712/138).

**Table I:** Demographic data at baseline and during infection

Subjects	N	Sex (M/F)	Age (y)∗	Smoking (pack-years)∗	FEV_1_ (% of predicted)∗		FEV_1_/FVC (%)∗		Peak sputum virus load (log_10_ copies/mL)
					Baseline	Day 9	Baseline	Day 9	
Nonsmoker	11	4/7	62 ± 5.1†	0 ± 0	101 ± 11.8	89 ± 10.8	78 ± 3.7	87 ± 9.0	7 ± 2.4
Smoker	20	10/10	51 ± 7.1	34 ± 10.5	104 ± 14.5	89 ± 26.1	79 ± 5.7	73 ± 9.0	6 ± 3.6
COPD	17	12/5	61 ± 8.1†	46 ± 21.0‡	68 ± 5.1§	58 ± 11.7§	57 ± 8.1§	54 ± 10.1§	8 ± 3.9

*FVC*, Forced vital capacity.

∗ Results are expressed as mean ± SD.

† *P* = .0003 vs smokers (Student *t* test).

‡ *P* = .025 vs smokers (Student *t* test).

§ *P* < .0001 vs nonsmokers and smokers.

### Experimental infection with RV-16 and confirmation of infection

Ten 50% tissue culture infective doses (10 TCID_50_) of RV-16 were administered on day 0 by nasal spray as previously described.^4^^,^^19^ RV infection was confirmed by at least 1 of the following: positive nasal lavage, sputum or bronchoalveolar lavage standard or quantitative PCR for RV, positive culture of RV-16, or seroconversion defined as a titer of serum-neutralizing antibodies to RV-16 of at least 1:4 at 6 weeks as described.^4^^,^^19^

### Blood and sputum inflammatory markers

Peripheral blood eosinophils were counted at baseline and on day 7 after infection. Sputum was sampled at baseline and on days 3, 5, 9, 12, 15, 21, and 42 during/postinfection. Details of sputum processing are provided in previous publications.^4^^,^^19^ Sputum eosinophils in cytospin were counted and mediators eotaxin, eotaxin-3, IL-4, IL-5, CXCL8/IL-8, IL-1β, and TNF were measured using the Mesoscale Discovery platform (Meso Scale Diagnostics, Rockville, Md).^19^ Eosinophilic cationic protein, pentraxin3, cathelicidin (LL-37), and neutrophil elastase were measured using ELISA kits according to the manufacturer’s instructions.^20^

#### Bronchoscopy and clinic data

Bronchial biopsies were taken approximately 14 days before infection (baseline), on day 7 during infection (acute infection) in all subjects, and at 42 days after infection (convalescence) in 11 subjects with COPD and 12 smoking controls.

### Immunohistochemistry

Neutrophil elastase^+^ neutrophils, EG2^+^ eosinophils, tryptase^+^ mast cells, CD4^+^ and CD8^+^ T lymphocytes and CD20^+^ B lymphocytes, and CD68^+^ monocytes/macrophages were immunostained as previously described.^6^

### Quantification

In slides coded to avoid observer bias, the areas of epithelial (epi) and subepithelial (sub) were assessed using an Apple Macintosh computer and Image 1.5 software (National Institute of Mental Health, Bethesda, Md). Inflammatory cells were seen and counted by light microscope. The data for cell counts were expressed as the number of positive cells per mm^2^ of the subepithelium and per 0.1 mm^2^ of the epithelium.

### Statistical analysis

One-way ANOVA followed by the unpaired Student *t* test was used for the analyses of age, smoking pack-years, and lung function data between groups. In respect of cell counts in blood, sputum, and biopsies and mediators in sputum, these data were nonnormally distributed and overall differences between all groups and between 3 time points within group were assessed first using the Kruskal-Wallis test, which, if significant, was followed by Wilcoxon matched pairs test for within-group differences between baseline and infection. The between-group differences were analyzed by Mann-Whitney tests. Spearman rank correlation was used for correlations between the numbers of inflammatory cells and virus load/physiologic/clinical data/sputum inflammatory markers/blood eosinophils. A *P* value of less than .05 was accepted as statistically significant. Further details of the methods are provided in this article’s Methods section in the Online Repository at www.jacionline.org.

## Results

### Histology

**Fig 1:**
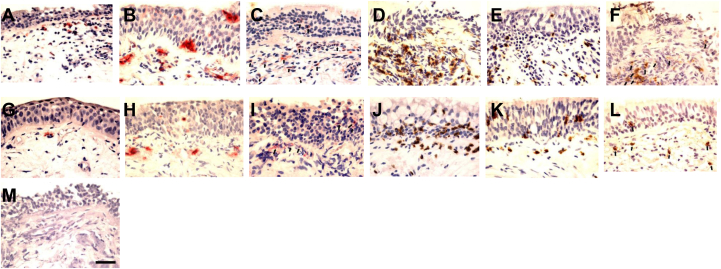
Immunohistochemistry-stained cells are seen as red fuchsin or brown diaminobenzidinen positivity: RV-16 infection on day 7 increased numbers of (**A**) eosinophils, (**B**) neutrophils, (**C**) CD68^+^ (*arrows*), (**D**) CD8^+^, (**E**) CD4^+^, and (**F**) CD20^+^ (*arrows*) cells in bronchial mucosa of subjects with COPD compared with their baseline numbers of (**G**) eosinophils, (**H**) neutrophils, (**I**) CD68^+^ (*arrows*), (**J**) CD8^+^, (**K**) CD4^+^, and (**L**) CD20^+^ (*arrows*) cells. **M,** Negative control shows an absence of signal (internal scale bar = 20 μm for all).

Inflammatory cells were present in both the bronchial epi- and sub-compartments. Representative photographs are depicted in Fig 1 (*A-M*). EG2^+^ eosinophils (Fig 1, *A*), elastase^+^ neutrophils (Fig 1, *B*), CD68^+^ monocytes/macrophages (Fig 1, *C*), CD4^+^ (Fig 1, *D*), and CD8^+^ (Fig 1, *E*). T lymphocytes and CD20^+^ (Fig 1, *F*) B lymphocytes appeared to be more frequent in the bronchial mucosa of patients with COPD on day 7 postinfection compared with their own baselines (Fig 1, *G-L*). Application of irrelevant antibody for the inflammatory cell markers was negative (Fig 1, *M*).

### Subepithelial inflammatory cells are increased from baseline to postinfection in patients with COPD

**Fig 2:**
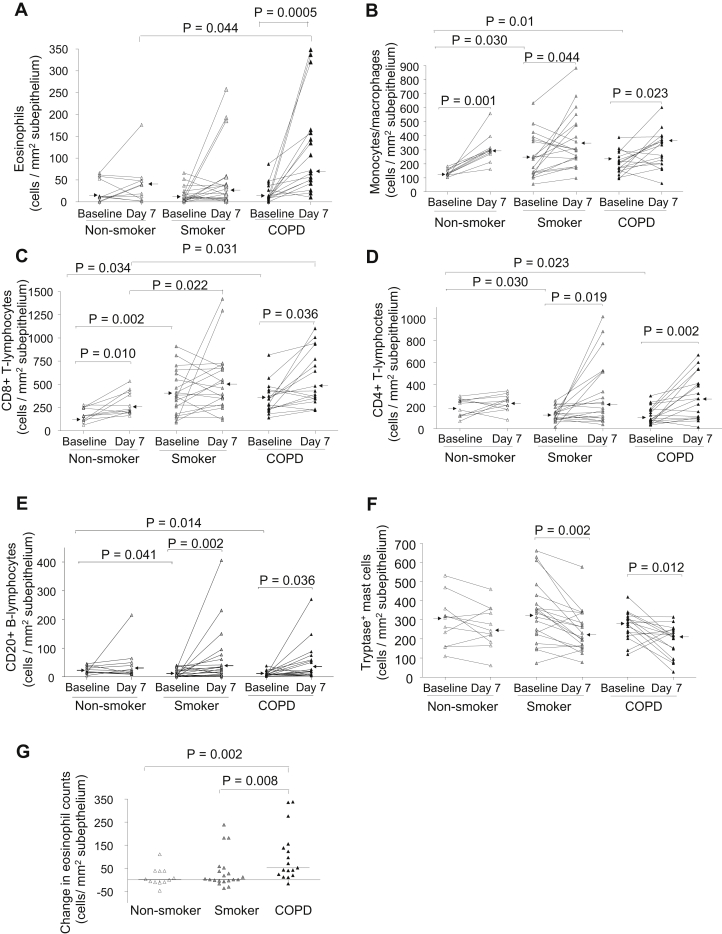
Counts for subepithelial (**A**) eosinophils, (**B**) CD68^+^ monocytes/macrophages, (**C**) CD8^+^ and (**D**) CD4^+^ T lymphocytes, (**E**) CD20^+^ B lymphocytes, and (**F**) tryptase^+^ mast cells in bronchial biopsies of healthy nonsmokers, healthy smokers, and subjects with COPD at baseline and day 7 after RV-16 infection. The data are expressed as the number of positive cells per mm^2^ of sub. **G,** Changes in counts of subepithelial eosinophils from baseline to day 7 postinfection in bronchial biopsies of healthy nonsmokers, healthy smokers, and subjects with COPD. The data are expressed as change in the number of eosinophils per mm^2^ of sub. Triangles show individual counts, and arrows/horizontal bars show median values (Wilcoxon matched pairs test and Mann-Whitney *U* test).

The most striking increase in absolute cell counts on day 7 postinfection compared with baseline in patients with COPD was a greater than 6-fold increase in numbers of sub-eosinophils (*P =* .0005, Fig 2, *A*, and Table II). On day 7, the numbers of sub-eosinophils in the subjects with COPD was significantly higher compared with those in nonsmokers (*P* = .044). In subjects with COPD, there was a nonsignificant trend for an increase in sub-neutrophils (*P =* .087, Table II). The numbers of sub-CD68^+^ cells were significantly increased on day 7 postinfection from baseline in all 3 groups (*P* = .001-.044, Fig 2, *B*). Sub-CD8^+^ cells increased significantly on day 7 from baseline in the COPD and nonsmoker groups (*P* = .036 and .010, respectively, Fig 2, *C*). Sub-CD8^+^ counts in subjects with COPD and smokers were significantly higher compared with those in nonsmokers on day 7 (*P* = .031 and .022, respectively, Fig 2, *C*). Sub-CD4^+^ and CD20^+^ counts significantly increased on day 7 from baseline in COPD and smoker groups (*P* = .002-.041, Fig 2, *D* and *E*). The elevated numbers of sub-neutrophils and CD8^+^ cells in COPD groups persisted at week 6, remaining at similar median levels to their counts at day 7 (Table II), whereas sub-eosinophils, CD68^+^, CD4^+^, and CD20^+^ cells had returned to their respective baseline levels (Table II). Sub-tryptase^+^ mast-cell counts were significantly decreased from baseline to day 7 postinfection in the smoker and COPD groups (*P* = .002 and .012, respectively, Fig 2, *F*) and also decreased from baseline to week 6 in the COPD group (*P* = .049, Table II).

**Table II:** Counts of phenotype of inflammatory cells infiltrating in epithelial and subepithelial areas in bronchial mucosa of subjects with COPD and control subjects

Groups	COPD			Healthy smokers			Healthy nonsmokers	
	Baseline (N = 17)	Day 7 (N = 17)	Week 6 (N = 11)	Baseline (N = 20)	Day 7 (N = 20)	Week 6 (N = 12)	Baseline (N = 11)	Day 7 (N = 11)
Epi∗								
Neutrophil elastase^+^	4 (1-18)	8† (1-46)	14† (2-47)	3 (0-30)	8 (0-36)	10 (0-47)	4 (16-0.4)	4 (0-24)
EG2^+^	0 (0-6)	0.4 (0-11)	0.4 (0-12)	0.2 (0-8)	0.4 (0-31)	0.3 (0-5)	0 (0-19)	0.7 (0-68)
Tryptase^+^	3 (0-16)	3 (0-8)	4 (0-14)	3 (0-35)	3 (0-21)	11 (0-25)	2 (0-9)	3 (0-16)
CD68^+^	11 (1-61)	18 (1-86)	31 (4-56)	15 (1-51)	24†‡ (5-88)	13 (0-77)	8 (3-27)	14 (4-22)
CD4^+^	3 (0-14)	8† (0-96)	5 (2-70)	3 (0-13)	6†§ (1-39)	2 (0-18)	4 (0-11)	8† (2-44)
CD8^+^	55‖ (31-183)	67‡ (27-178)	94 (39-253)	67‖ (7-187)	72‡ (30-165)	73 (20-116)	38 (9-72)	321 (15-96)
CD20^+^	0 (0-0.3)	1† (0-19)	0 (0-2)	0 (0-3)	2† (0-5)	0 (0-4)	0 (0-2)	0.5† (0-6)
Sub∗								
Neutrophil elastase^+^	109 (40-337)	135 (64-445)	155 (72-201)	135 (22-492)	148 (27-564)	117 (18-289)	125 (44-295)	149 (67-469)
EG2^+^	11 (0-87)	72† (11-349)	23 (2-169)	11 (0-66)	25 (1-257)	34 (0-199)	12 (0-66)	38 (1-176)
Tryptase^+^	301 (119-480)	220† (72-347)	184† (140-323)	341 (73-661)	220† (77-576)	300 (15-605)	310 (109-531)	248 (61-459)
CD68^+^	201‖ (95-388)	334† (61-603)	228 (138-379)	234‖ (55-633)	302† (97-883)	217 (125-506)	127 (102-182)	294† (161-559)
CD4^+^	66‖ (37-296)	221† (12-666)	167 (75-406)	142‖ (14-251)	226† (36-1014)	83 (29-447)	199 (67-293)	230 (32-342)
CD8^+^	243‖ (142-816)	429†‡ (148-1003)	408† (311-593)	376‖ (90-910)	491‡ (117-1420)	541 (31-736)	159 (63-278)	236† (163-533)
CD20^+^	9‖ (2-44)	23† (4-270)	13 (6-138)	9‖ (0-39)	29† (0-406)	16 (0-51)	18 (5-46)	20 (9-215)

∗ Values are medians (ranges) of positive cell counts per 0.1 mm^2^ epi and per mm^2^ sub.

† *P* = .0005-.044 vs their own baselines, respectively.

‡ *P* = .011-.049 vs nonsmoker day 7, respectively.

§ *P* = .031 vs its own week 6.

‖ P = .0005-0.047 vs nonsmoker baseline.

### Epithelial inflammatory cells are increased from baseline to postinfection in patients with COPD

**Fig 3:**
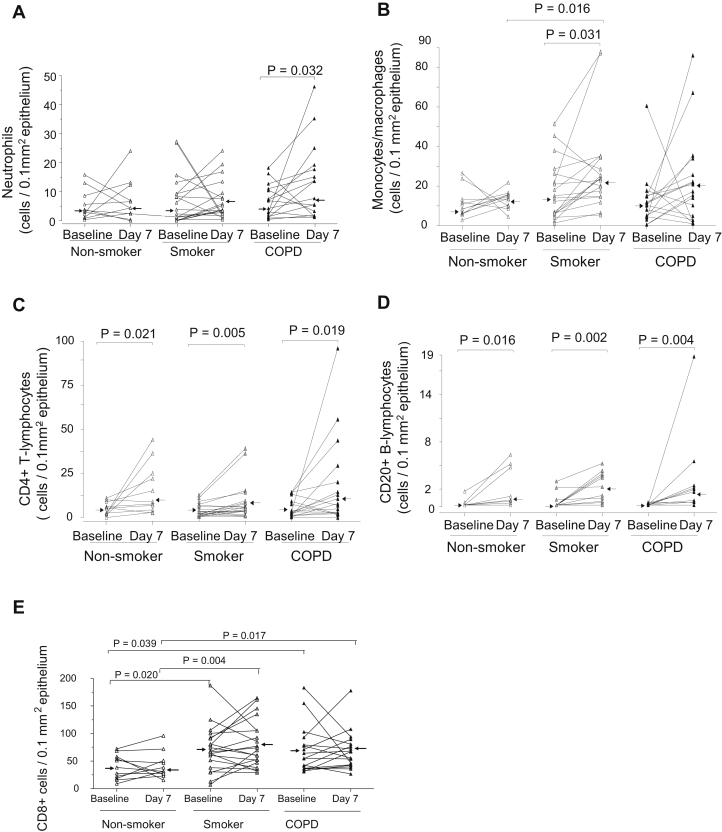
Counts for epithelial (**A**) neutrophils, (**B**) CD68^+^ monocytes/macrophages, and (**C**) CD4^+^, (**D**) CD20^+^, and (**E**) CD8^+^ lymphocytes in bronchial biopsies of healthy nonsmokers, healthy smokers, and subjects with COPD at baseline and day 7 after RV-16 infection. The data are expressed as the number of positive cells per 0.1 mm^2^ of epi. Triangles show individual counts, and arrows show median values (Wilcoxon matched pairs test and Mann-Whitney *U* test).

Compared with baseline, there was a significant increase in numbers of epi-neutrophils at day 7 postinfection in the COPD group only (*P* = .032, Fig 3, *A*, and Table II) and epi-neutrophils remained significantly higher (*P* = .005) than baseline level at week 6 postinfection (Table II). The numbers of epi-CD68^+^ cells in smokers were significantly increased on day 7 from baseline (*P* = .031, Fig 3, *B*). Also, on day 7, epi-CD68^+^ cell counts in the smokers were significantly higher than those in the nonsmokers (*P* = .016). The numbers of epi-CD4^+^ and CD20^+^ cells increased significantly from baseline to day 7 postinfection in all 3 groups (*P* = .002-.021, Fig 3, *C* and *D*). The numbers of epi-CD8^+^ cells on day 7 in the smokers and subjects with COPD were significantly higher compared with the numbers in the nonsmoker group (*P* = .004 and .017, respectively, Fig 3, *E*). The elevated numbers of epi-CD8^+^ cells in the smoker and COPD groups persisted at week 6, remaining at similar levels to their counts at day 7 (Table II). There were no significant differences in counts of epi-eosinophils and mast cells between baseline and infection in any subject group.

### Baseline CD4^+^ T lymphocytes and CD20^+^ B lymphocytes in smoker and COPD groups are decreased compared with the healthy nonsmoker group

The baseline numbers of sub-CD68^+^ and both epi- and sub-CD8^+^ cells were significantly higher (*P* = .002-.039, Fig 2, *B* and *C*, and Fig 3, *E*, Table II), whereas those of sub-CD4^+^ and CD20^+^ cells were significantly lower (*P* = .014-.041, Fig 2, *D* and *E*, Table II) in smokers and subjects with COPD compared with the baseline of nonsmokers.

### Greater magnitude of increase in eosinophils in the COPD group postinfection

To investigate differences in inflammatory responses of nonsmokers, smokers, and subjects with COPD to RV infection, the magnitude of the changes in inflammatory cell counts from baseline to infection was compared between groups. The change in numbers of sub-eosinophils from baseline to day 7 postinfection in subjects with COPD was significantly greater than the changes in both the nonsmokers and smokers (*P* = .002 and .008, respectively, Fig 2, *G*), with 16 of 17 subjects with COPD experiencing an increase during exacerbation, with a median increase of 57 eosinophils/mm^2^ of sub in subjects with COPD versus 1 in nonsmokers and 3 in smokers. In contrast, there were no significant differences between groups in changes from baseline to day 7 for any other phenotype inflammatory cells.

### Blood and sputum eosinophils in subjects with COPD postinfection

**Fig 4:**
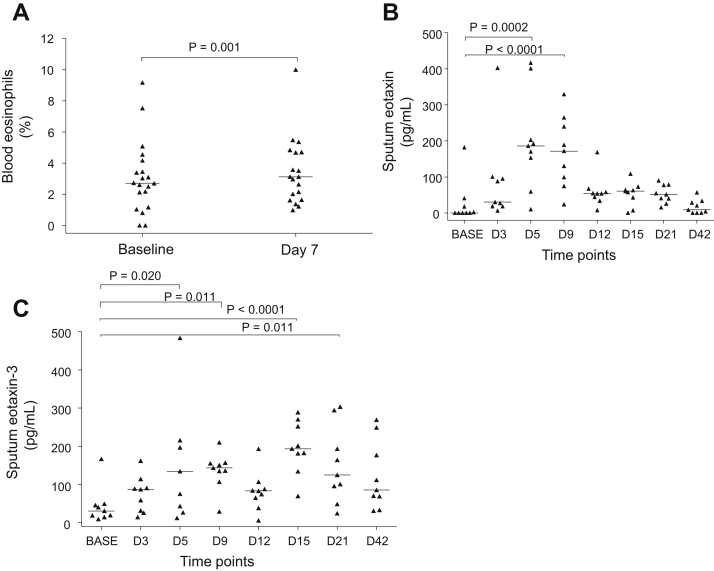
Blood eosinophils and sputum eosinophil-related soluble mediators in subjects with COPD during experimental RV infection: (**A**) blood eosinophil percentages at baseline and day 7 postinfection and (**B**) eotaxin and (**C**) eotaxin-3 in induced sputum at baseline and day 3 to 42 postinfection. Triangles show individual counts, and horizontal bars show median values (Wilcoxon matched pairs test). *BASE*, Baseline.

There was no change in blood eosinophil numbers between baseline and after infection in any subject group; however, there was a small but statistically significant increase in blood eosinophil percentages in the subjects with COPD from baseline to day 7 (2.72% vs 3.13%, *P* = .001, Fig 4, *A*) but not in the control subjects. We have previously reported no significant increase in sputum eosinophils when the 2 studies were analyzed separately.^4^^,^^19^ When the 2 studies were combined herein, again there was no significant increase from baseline in either sputum eosinophil numbers or percentages on any day after infection in the subjects with COPD. There were no correlations between mucosal eosinophils and blood or sputum eosinophils.

### Sputum inflammatory markers

We measured chemokines/cytokines relevant to eosinophil biology in sputum in a subset of the subjects with sufficient sputum supernatants remaining. Following infection there were significant increases in eotaxin (*P* = .0002 and <.0001, Fig 4, *B*) and eotaxin-3 (*P* < .00001-.020, Fig 4, *C*) in the subjects with COPD but not in the controls without COPD (data not shown). There were no significant increases in IL-4, IL-5, or eosinophilic cationic protein following infection (data not shown). There were no correlations between mucosal eosinophils and any of these sputum markers.

### Associations between mucosal inflammatory cell numbers and virus load/clinical outcomes and smoking pack-years

**Fig 5:**
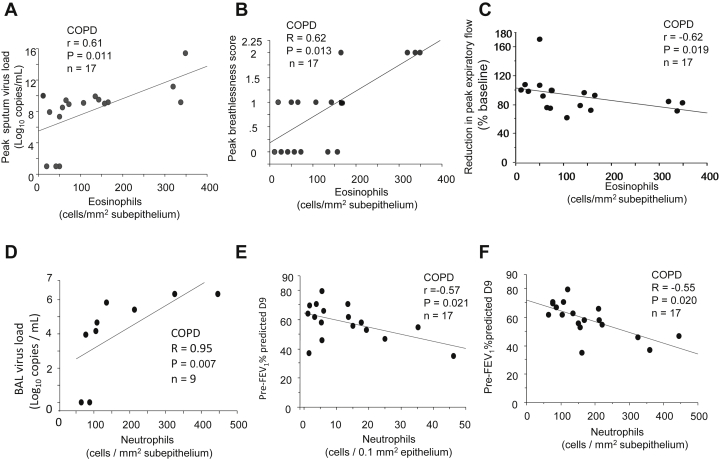
Correlations, in subjects with COPD, between the numbers of subeosinophils on day 7 postinfection and (**A**) peak sputum virus load, (**B**) peak breathlessness scores, and (**C**) reduction in peak expiratory flow (% fall from baseline), recorded on day 9 postinfection, (**D**) between BAL virus load and subneutrophils on day 7 postinfection; between counts of (**E**) epi- and (**F**) sub-neutrophils on day 7 and prebronchodilator FEV_1_% predicted at day 9 (Spearman rank correlation, n = 17 or 9). *BAL*, Bronchoalveolar lavage.

The numbers of sub-eosinophils in subjects with COPD during infection were associated with peak sputum virus load (*r* = 0.61, *P* = .011, Fig 5, *A*) and also with COPD exacerbation severity because sub-eosinophils on day 7 were related to peak breathlessness scores (*r* = 0.62, *P* =.013, Fig 5, *B*) and to reductions in peak expiratory flow (*r* = −0.62, *P* = .019, Fig 5, *C*) during infection.

**Figure E1:**
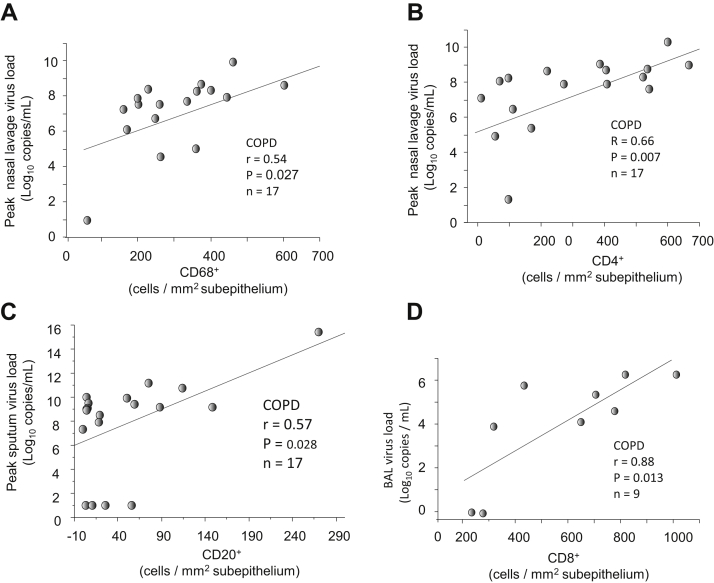
In subjects with COPD, correlations between the numbers of (**A**) sub-CD68^+^ and (**B**) CD4^+^ on day 7 and peak nasal lavage virus load, (**C**) between sub-CD20^+^ on day 7 and peak sputum virus load, and (**D**) between sub-CD8^+^ cells on day 7 and BAL virus load (Spearman rank correlation, n = 17 or 9).

**Figure E2:**
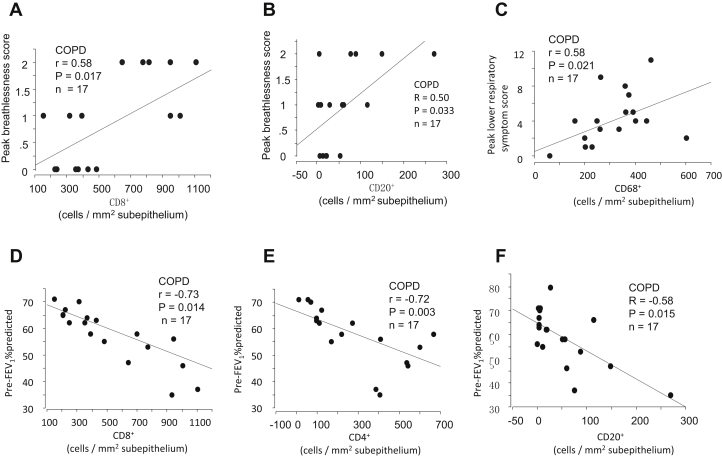
In subjects with COPD, correlations between the numbers of (**A**) sub-CD8^+^ and (**B**) CD20^+^ cells on day 7 postinfection and peak breathlessness scores, (**C**) between counts of sub-CD68^+^ cells and peak lower respiratory tract symptom score, and between the numbers of (**D**) sub-CD8^+^, (**E**) CD4^+^, and (**F**) CD20^+^ cells on day 7 and prebronchodilator FEV_1_% predicted at day 9 (Spearman rank correlation, n = 17).

In subjects with COPD, sub-neutrophils correlated with bronchoalveolar lavage virus load on day 7 (*r* = 0.95, *P* = .007, Fig 5, *D*) and higher numbers of both epi- and sub-epithelial neutrophils were significantly associated with lower prebronchodilator FEV_1_% predicted on day 9 (*r* = −0.57 and −0.55, *P* = .021 and .020, respectively, Fig 5, *E* and *F*). Mucosal CD68^+^ monocytes/macrophages and lymphocytes during infection were also related with virus load, clinical symptom severity, and reductions in lung function during infection, which are presented in the Results section and Fig E1 (*A-D*) and Fig E2 (*A-F*) in this article’s Online Repository at www.jacionline.org.

**Figure E3:**
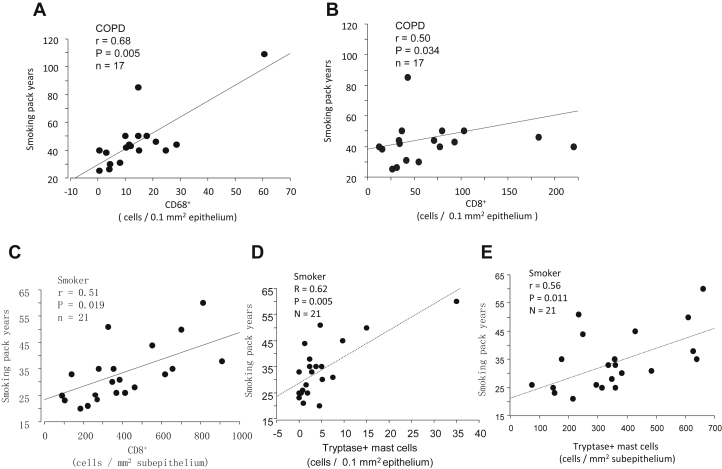
Correlations between smoking pack-years and numbers of (**A**) baseline epi-CD68^+^ and (**B**) CD8^+^ cells in subjects with COPD and (**C**) baseline sub-CD8^+^ cell counts, (**D**) epi- and (**E**) sub-tryptase+ mast-cell counts in healthy smokers (Spearman rank correlation, n = 17 for COPD and n = 21 for healthy smokers).

At baseline, the counts of epi-CD68^+^ and CD8^+^ cells in subjects with COPD and sub-CD8^+^ cells and both epi- and sub-tryptase+ mast cells in smokers correlated positively with smoking pack-years (*r* = 0.5-0.68, *P* = .005-.034, Fig E3, *A-E*).

### Correlations between mucosal eosinophil cell numbers and sputum inflammatory markers

**Fig 6:**
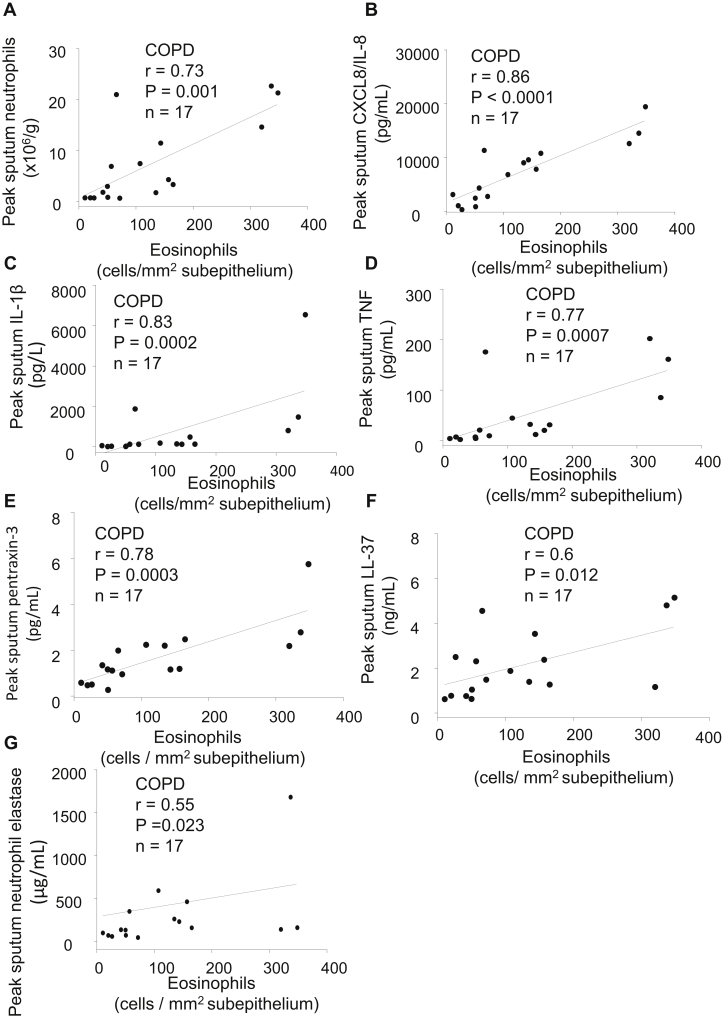
Correlations, in subjects with COPD, between the numbers of subepithelial eosinophils at day 7 postinfection and peak sputum: (**A**) neutrophils, (**B**) CXCL8/IL-8, (**C**) IL-1β, (**D**) TNF, (**E**) pentraxin-3, (**F**) LL-37, and (**G**) neutrophil elastase (Spearman rank correlation, n = 17 for all).

We finally examined the relationships between sub-eosinophil numbers on day 7 postinfection and sputum inflammatory markers previously measured in the subjects with COPD.^4^^,^^19^ Sub-eosinophils correlated with peak sputum neutrophils (*r* = 0.73, *P* = .001, Fig 6, *A*), but there was no significant correlation between sub-eosinophils and peak sputum eosinophils. Sub-eosinophils also correlated strongly with peak values during infection of several sputum inflammatory mediators and antimicrobial peptides including CXCL8/IL-8 (*r* = 0.86, *P* < .0001, Fig 6, *B*), IL-1β (*r* = 0.83, *P* = .0002, Fig 6, *C*), TNF (*r* = 0.77, *P* = .0007, Fig 6, *D*), pentraxin-3 (*r* = 0.78, *P* = .0003, Fig 6, *E*), LL-37 (*r* = 0.6, *P* = .012, Fig 6, *F*), and neutrophil elastase (*r* = 0.55, *P* = .023, Fig 6, *G*). However, there were no correlations between epi- or sub-neutrophils and the sputum inflammatory markers.

## Discussion

We have found that experimental RV infection induced eosinophils and neutrophils only in the subjects with COPD, whereas macrophages and T and B lymphocytes were increased in both subjects with COPD and control subjects. Statistically significant positive associations were found between inflammatory cell numbers and virus load, respiratory symptom severity, and reductions in lung function in subjects with COPD. The numbers of sub-eosinophils also correlated with inflammatory markers in sputum.

### Eosinophils and neutrophils

The presence and role of eosinophils in COPD exacerbations have remained unclear, with conflicting results from studies using sputum. Some studies have reported increased numbers of eosinophils at exacerbation but did not include virus detection.^20^^,^^21^ Bafadhel et al^14^ reported that there were only 3% of exacerbations where virus and sputum eosinophil coexisted. We reported increased sputum eosinophils restricted to virus-induced severe exacerbations.^13^ Others found no significant increase in eosinophil numbers in virus-induced exacerbations.^22^ These discrepancies may be due to heterogeneity in the etiology of COPD exacerbations,^23^ timing of sampling, effects of treatment, and variation in disease severity.^24^ Studies using bronchial biopsies have reported increased eosinophils in the bronchial mucosa of naturally occurring exacerbations,^16^^,^^18^^,^^25^ but the role of viruses as a cause of such mucosal eosinophilia remains uncertain. Our present study is the first to compare the effects of experimentally administered virus on the bronchial mucosal inflammatory response using bronchial biopsies from the same subjects when stable and during exacerbations in treatment-naive, nonintubated subjects with COPD. A significant increase in mucosal, but not sputum, eosinophils was demonstrated only in the subjects with COPD following RV infection. Also, the change in sub-eosinophil counts (not for other cell types) from baseline to day 7 postinfection in subjects with COPD was significantly greater than those in nonsmoker and smoker control subjects. This demonstrated a clear difference in the mucosal inflammatory response between subjects with and without COPD. Moreover, greater numbers of sub-eosinophils were associated with greater virus load, more symptoms, bigger falls in lung function, and higher sputum inflammatory markers. The findings of RV-induced eosinophilia are noteworthy given that they were observed in subjects with relatively mild COPD who had no history of asthma or allergic rhinitis and who tested negative to 10 aeroallergens on skin prick tests. The data support a pathogenic role for bronchial mucosal eosinophilia in RV infection–induced COPD exacerbations. Therefore, in exacerbations of COPD where eosinophils are identified and steroid^26^ or anti–IL-5 eosinophil-targeting^27^^,^^28^ therapies are considered, the addition of future novel antiviral therapies may be of particular benefit. In addition, blood eosinophils have been examined as a marker to guide corticosteroid use in COPD exacerbations,^29^^,^^30^ though this approach continues to be debated.^31^^,^^32^ Our data suggest that the relationship between blood, sputum, and mucosal eosinophils is complex. The lack of a relationship between blood and mucosal eosinophils implies that using blood eosinophils alone as a marker of airway mucosal eosinophilia may result in some patients without blood eosinophilia not receiving corticosteroids when there is, indeed, mucosal eosinophilia.

Contrary to the results seen with eosinophils, sub-neutrophils were not significantly increased whereas epi-neutrophils were increased in subjects with COPD, when higher numbers were positively related to virus load and falls in lung function. We have also reported previously that neutrophils are significantly increased in the sputum of these subjects with COPD,^4^^,^^19^ with strong correlations between sputum neutrophils and sputum neutrophil elastase, IL-1β, TNF, CXCL8/IL-8, pentraxin-3, and LL-37.^19^^,^^33^ Surprisingly, in our present analyses, these sputum markers correlated better with sub-eosinophils than with epi/sub-neutrophils. These data suggest that virus infection induces an innate inflammatory response involving mediators such as IL-1β, TNF, and CXCL8/IL-8 that contribute to the recruitment of both neutrophils and eosinophils. It is considered that neutrophils transit rapidly from blood through the bronchial mucosal into the airway lumen and thus their numbers in sputum reflect mucosal tissue neutrophilic inflammation. In contrast, it is likely that eosinophils transit more slowly and are retained in the mucosal compartment. Thus, we speculate that the contribution of eosinophils may well be underestimated in studies using sputum alone. Moreover, therapies targeting eosinophils have focused on the T_H_2 pathway in both asthma^34^^,^^35^ and COPD.^27^^,^^28^ In distinction to asthma, our present data in COPD show associations between eosinophils and mediators of innate inflammation, suggesting that other pathways may be involved in eosinophil recruitment to the airways, at least in the context of acute viral infection.

### Lymphocytes, macrophages, and mast cells

Earlier studies have suggested a pathogenic role for CD68^+^ monocytes/macrophages and CD8^+^ T cells in COPD^6^^,^^8^^,^^36^^,^^37^ but the mechanisms of their increased recruitment in COPD are not well known. A previous study has demonstrated a positive correlation between the number of bronchial mucosal CD8^+^ cells in subjects with COPD and the number of pack-years smoked.^7^ Here, we have confirmed that baseline numbers of CD68^+^ and CD8^+^ cells are significantly greater in smokers and in subjects with COPD than in nonsmokers and that baseline CD68^+^ and CD8^+^ counts in subjects with COPD correlate positively with smoking pack-years. In addition, for the first time, we present data showing that CD8^+^ T cells are increased in nonsmokers and those with COPD from baseline following infection but not in the smokers who had significantly higher baseline CD8^+^ counts compared with nonsmokers at baseline. In contrast, RV infection induced increases in CD68^+^ cells in all 3 groups. The numbers of CD8^+^ cells were significantly greater in smoker and COPD groups than in the nonsmoker group on day 7 postinfection. At 6 weeks, CD8^+^ T-cell numbers in both smoker and COPD groups were still increased. These data indicate that smoking and virus infection have an additive and prolonged effect on the pulmonary recruitment of CD8^+^ cytotoxic T cells.

Previously we have demonstrated that CD4^+^ cells are significantly fewer in subjects with COPD in its stable phase compared with nonsmoker controls.^18^ However, at that time a healthy smoker group was not available for comparison. Gosman et al^38^ have reported an increase in bronchial mucosal B lymphocytes in subjects with Global Initiative for Chronic Obstructive Lung Disease stage II and III COPD compared with healthy smokers. Hogg et al^9^ reported that the accumulated volume of B cells in small airways was increased in stage III and IV COPD and the increasing number of B cells was associated with increasing severity of COPD. But in the last study a healthy nonsmoker group was not included, and the presence or absence of the virus infection was not investigated in either of the aforementioned studies. Therefore, the roles of smoking and virus infection in CD4^+^ and CD20^+^ cell recruitment into the bronchial mucosa remain unclear. Herein, we report for the first time that both smokers and subjects with COPD have lower numbers of baseline sub-CD4^+^ and CD20^+^ cells compared with nonsmokers at baseline whereas RV infection recruited CD4^+^ and CD20^+^ cells into bronchial mucosa in all 3 groups. These findings indicate that smoking per se increases CD68^+^ and CD8^+^ cells and decreases CD4^+^ and CD20^+^ cells, whereas RV infection increases the recruitment of all these cell types in the bronchial mucosa of all subjects.

Finally, we consider that the reduction in the number of sub-mast cells is likely due to infection-induced degranulation, leading to fewer cells containing sufficient tryptase to stain positive for the purpose of their identification. The effects of smoking and virus infection on mast-cell biology in COPD exacerbations require further study.

### Study limitations

Our subjects had relatively mild Global Initiative for Chronic Obstructive Lung Disease stage II COPD, and we suggest that in a more severe COPD population eosinophilic inflammation may be even more prominent. We acknowledge that our group sizes were relatively small, particularly for those where we had sputum eosinophil mediators: thus, significant correlations may have been missed. Furthermore, this is an exploratory and hypothesis-generating study and as such we did not control for type I errors arising from multiple comparisons. As a result, the observed significant differences and associations may be subject to false positives. Further hypothesis-testing studies are needed to confirm selected of our observations. However, the relative homogeneity of subjects allowed for more reliable interpretation of the data, which is difficult to obtain in naturally occurring exacerbations of COPD.

### Conclusions

Experimental RV infection increases the numbers of bronchial mucosal eosinophils and neutrophils only in subjects with COPD, whereas monocytes/macrophages, CD8^+^ and CD4^+^ T lymphocytes, and CD20^+^ B lymphocytes increased in both subjects with COPD and controls without COPD. The eosinophilic inflammatory response to RV infection in the bronchial mucosa of subjects with COPD differs from that seen in the airway lumen and in blood. The increased numbers of inflammatory cells in subjects with COPD correlated with virus load and illness severity, and eosinophils also associated with sputum innate inflammatory mediators during the infection. In addition, chronic cigarette smoking decreased the numbers of CD4^+^ and CD20^+^ cells and increased the numbers of CD8^+^ and CD68^+^ cells. Thus, our findings provide new insights into previously undescribed patterns of inflammatory response that occur during experimental RV-induced exacerbations of COPD and also smoking per se: these data could have an impact on the design of future treatment modalities.

**Key messages**

• Experimental RV infection increases bronchial mucosal eosinophils and neutrophils in subjects with COPD only, and macrophages and lymphocytes in both subjects with COPD and controls without COPD.
• RV-induced bronchial mucosal inflammation is associated with illness severity during virus-induced COPD exacerbations.
• Antiviral and anti-inflammatory therapies could attenuate bronchial inflammation and ameliorate virus-induced COPD exacerbations.

## Figures and Tables

**Table E1:** Inclusion criteria for study subjects

All subjects • Age 40-75 y • No history of asthma or allergic rhinitis and not atopic on skin testing • Absence of a current or previous history of bronchiectasis, carcinoma of the bronchus, or other significant respiratory disease (other than COPD) • Absence of significant systemic disease • No COPD exacerbation or respiratory tract infection within the previous 8 wk • Serum antibodies to RV-16 at screening in a titer <1:2 • No treatment with antibiotics, oral, inhaled, or nasal topical steroids, long-acting β-agonists, or tiotropium in the previous 3 mo
COPD group • FEV_1_ 50%-79% predicted normal value and β-agonist reversibility <12% • FEV_1_/FVC < 70% • Current or ex-smokers with at least 20 pack-years cumulative smoking
Smokers • FEV_1_ ≥ 80% predicted normal value • FEV_1_/FVC > 70% • Current or ex-smokers with at least 20 pack-years cumulative smoking
Nonsmokers • FEV_1_ ≥ 80% predicted normal value • FEV_1_/FVC > 70% • Nonsmokers

*FVC*, Forced vital capacity.

## Methods

### Participants

The subjects were recruited to 2 experimental studies that have been published previously, study 1^E1^ and study 2.E2, E3 The first study ran from 2003 to 2007: 2 groups of study subjects—13 smokers with COPD and 13 smokers with normal lung function were recruited initially.^E1^ The second study ran from 2007 to 2010: separate cohorts of 18 smokers with COPD, 15 smokers without airway obstruction, and an additional control group of 19 healthy nonsmokers with normal lung function were enrolled.E2, E3 The inclusion criteria presented in Table E1 were same for the 2 smoker groups in the 2 studies. All subjects were nonatopic as defined by negative reactions to a 6-grass pollen mix, house-dust mite, cat, dog, *Aspergillus fumigatus*, *Cladosporium herbarium*, *Alternaria alternata*, birch pollen, 3 tree pollen mix, and nettle pollen (ALK Abello, Reading, United Kingdom [UK]) on skin prick tests, and none had any history of asthma or allergic rhinitis. The protocols for the 2 studies were identical. A total of 31 subjects with COPD, 28 smokers, and 19 nonsmokers were inoculated with low-dose RV-16 and 77 of 78 subjects completed the study; 1 subject with COPD withdrew because of ill health believed unconnected to the study.^E1^ RV infection was confirmed in 20 of 30 subjects with COPD (66.7%), 22 of 28 smokers (78.6%), and 11 of 19 nonsmokers (58%).E1, E2, E3 Only infected subjects were included in the present biopsy study. The bronchial biopsies were not obtained from 1 subject with COPD and 1 healthy smoker. Two day-7 biopsies obtained from 2 subjects with COPD and 1 day-7 biopsies from a smoker were of inadequate quality and excluded. Thus, actual data from 17 subjects with COPD, 20 healthy smokers, and 11 healthy nonsmokers (48 pairs of baseline and day 7 bronchial biopsies) were analyzed.

Subjects were recruited from a number of sources including newspaper advertisements, primary care, spirometry clinics, smoking cessation clinics, and outpatient hospital clinics. Initial screening visits suitability for the study was assessed and informed consent was obtained. All subjects had no respiratory tract infection within the previous 8 weeks. Their serum- neutralizing antibodies to RV-16 were measured^E1^ at screening and were in a titer of at least 1:2. Patients with COPD had no exacerbation and were not treated with oral, inhaled, or nasal topical steroids, long-acting β-agonists, or tiotropium in the previous 3 months. For those entering the study, baseline clinical sampling was performed 1 to 4 weeks before virus inoculation, which was at study day 0. Subjects were seen daily on the 9 days immediately after inoculation. The timeline for clinical measurements and sampling is outlined in the previous articles.E1, E2, E3

### Virus inoculation

Details regarding the preparation and safety testing of the RV-16 inoculum used in this study have been published.^E4^ The virus was diluted in a total volume of 1 mL of 0.9% saline and inoculated into both nostrils, using an atomizer (no. 286; DeVilbiss Co, Heston, UK).

### Blood and sputum

Both were collected at baseline and on days 5, 9, 12, 15, 21, and 42 postinoculation.E1, E3 Blood was also collected on day 7.

### Bronchoscopy

All bronchoscopies were carried out in the Endoscopy Unit at St Mary’s Hospital, Imperial College Healthcare NHS Trust by an experienced operator. Subjects were administered nebulized salbutamol (2.5 mg) and ipratropium bromide (0.5 mg) before the procedure and intravenous midazolam was used to provide sedation. Bronchoalveolar lavage (BAL) and bronchial biopsies were taken at baseline and day 7 postinfection for all subjects and 42 days (6 weeks, convalescence) in 12 smoking controls and 11 subjects with COPD using an Olympus bronchovideoscope BF, type 1T 10 with sterile FB 15C forceps (Olympus Co, Tokyo, Japan) from the subcarina of the basal segmental bronchi of the right lower lobe. Up to 3 biopsies were obtained from each subject and fixed immediately in 4% paraformaldehyde. Details of the collection and processing of nasal lavage, induced sputum, and BAL are provided previously.E1, E3

### Confirmation of RV-16 infection

RV infection was confirmed by at least 1 of the following: positive nasal lavage, sputum or BAL standard or quantitative PCR for RV, positive culture of RV-16, or seroconversion defined as a titer of serum-neutralizing antibodies to RV-16 of at least 1:4 at 6 weeks. Serology was performed at screening and 6 weeks postinfection by microneutralization test for neutralizing antibody to RV-16.^E5^ Virus was cultured by adding 250 μL of nasal lavage (from the day of peak virus load by quantitative PCR) to semiconfluent HeLa cells that were cultured for up to 5 passages. Cultured virus was confirmed as RV-16 by microneutralization assay with RV-16–specific antisera (American Type Culture Collection [ATCC]; titer 1:600).^E5^ RNA was extracted from samples (QIAamp viral RNA minikit; Qiagen Ltd, Crawley, UK) and reverse-transcribed (omniscript RT kit, Qiagen) with random hexamers. Standard RV PCR (PerkinElmer 9600 GeneAmp) was performed from 2 μL of cDNA in nasal lavage, an unprocessed plug of induced sputum, and unprocessed BAL.^E6^ The threshold of a positive virus infection is around 25 to 50 copies per microliter of cDNA and is based on 2-fold mean and plus 1 SD of the minimal detectable concentration, based on how the standard curve (double-stranded DNA) runs over many assays during the study period. To differentiate RV from other picornaviruses, BglI enzyme restriction digestion was carried out on the amplicons generated by RT-PCR.^E7^ Quantitative PCR was performed on 2 μL of cDNA to detect picornavirus using AmplitaqGold DNA polymerase (PE Biosystems ABI Prism 7700; Thermo Fisher Scientific UK Ltd, Leicestershire, UK).^E8^ A standard curve was produced by using serially diluted cloned product and results expressed as copies/mL. The sensitivity of this assay was 104 copies/mL. Virus load was measured with a real-time quantitative RT-PCR assay.^E9^

### PCR for other respiratory viruses

Infection with viruses other than RV was excluded by testing nasal lavage by PCR on random hexamer-primed cDNA for *Mycoplasma* and *Chlamydophila pneumoniae*, adenoviruses, respiratory syncytial virus, influenza AH1/AH3/B, parainfluenza 1–3, human metapneumoviruses, and coronaviruses 229E and OC43 as described,^E10^ except human metapneumovirus, which was adapted from Maertzdorf et al.^E11^

### Clinical procedures

Daily diary cards of upper and lower respiratory tract symptoms were commenced at screening and continued until 6 weeks after inoculation. Upper respiratory tract symptoms were measured using the Jackson scale assessing 8 symptoms—sneezing, headache, malaise, chilliness, nasal discharge, nasal obstruction, sore throat, and cough—graded 0 (absent) to 3 (severe).^E12^ The daily cold score was summated from the individual scores, and a clinical cold was defined using the Jackson criteria.^E12^ The scoring system for the lower respiratory tract symptoms of shortness of breath, cough, wheeze, sputum quantity, and sputum quality is shown in a previous article.^E13^ The daily lower respiratory tract score was summated from the individual scores, and a COPD exacerbation was defined as an increase in the lower respiratory tract score of at least 2 points over baseline for at least 2 consecutive days.E1, E14 For both upper and lower respiratory tract daily symptom scores, the mean scores on days −6 to 0 were calculated and subtracted from subsequent daily scores to correct for baseline symptoms.

### Pulmonary function

Spirometry was performed with a Micromedical MicroLab spirometer (MicroMedical, Rochester, UK) according to British Thoracic Society guidelines^E15^ before and 15 minutes after administration of salbutamol (200 mg) via a metered-dose inhaler and large-volume spacer for prebronchodilator and postbronchodilator values. The spirometry data were collected at baseline and on days 5, 9, 12, 15, 21, 28, 35, and 42 postinfection.

### Inflammatory markers

Peripheral blood eosinophils were counted at baseline and on day 7 after infection and in the Haematology laboratories of St Mary’s Hospital, Imperial College Healthcare NHS Trust. The ELISAs for detection of antimicrobial peptides and inflammatory mediators in sputum were carried out according to the manufacturers’ instructions and have been published previously.E1, E2 Briefly, plates were read on a Spectramax Plus 384 plate reader and the results read using SoftMax Pro software (Promega UK Ltd, Southampton, UK). Initial experiments were carried out to determine whether sample dilution was required. Experiments were carried out to determine the recovery of antimicrobial peptides from sputum. A sputum sample and PBS were spiked with the relevant protein at the same concentration and the levels detected compared. For all the proteins measured, the recovery in sputum was more than 80% that of the PBS sample. The sensitivities and sources of the individual ELISAs were as follows: pentraxin 3 (0.025 ng/mL; R&D Systems, Abingdon, UK); HBD-2 (8 pg/mL; PeproTech, London, UK); α-defensins 1-3 (156 pg/mL; Hycult Biotech, Cambridge, UK); cathelicidin LL-37 (0.1 ng/mL; Hycult Biotech), and neutrophil elastase (0.12 ng/mL; Immunodiagnostik, Benshein, Germany).

### Mesoscale Discovery

The mediators eotaxin, eotaxin-3, IL-4, IL-5, CXCL8/IL-8, IL-1β, and TNF in sputum were measured using the Mesoscale Discovery platform. The technique enables quantitative detection of between 1 and 9 mediators per well in a 96-well plate format using a Multi-spot technique, which has been published previously.^E3^ Briefly, the protocol requires addition of 25 μL blocking solution before incubation. Following plate washing, either sample or standard was added to the plate, followed by incubation and washing and addition of detection antibody. Finally, read buffer was added and the plate passed through the Sector imager for reading. The lower limits of detection of the individual analyses were as follows: eotaxin (10 pg/mL), eotaxin 3 (80 pg/mL) , IL-4 (0.1 pg/mL), IL-5 (0.3 pg/mL), CXCL8/IL-8 (0.6 pg/mL), IL-1β (1.17 pg/mL), and TNF (0.376 pg/mL).^E3^

### Immunohistochemistry

EnVision-alkaline phosphatase technique (Dako Ltd, Cambridge, UK) was used to label EG2^+^ eosinophils, neutrophil elastase^+^ neutrophils, tryptase^+^ mast cells, and CD68^+^ monocytes/macrophages. EnVision peroxidase staining method (Dako) was used to identify CD4^+^, CD8^+^ T lymphocytes, and CD20^+^ B lymphocytes. The immunostaining procedures for detecting the phenotypes of inflammatory cells were conducted by Techmate “Horizon” automated immunostainer (LJL Biosystems Inc, Sunnyvale, Calif) as previously described but with modification.^E16^ Irrelevant mouse IgG_1_ kappa antibody (MOPC21) was used to substitute for the primary layer as negative control for staining specificity of mouse mAbs. The following panel of monoclonal mouse antihuman antibodies (Dako) was applied to tissue sections: anti–neutrophil elastase (M0752), tryptase mast cell (M7052), CD4 (M0716), CD8 (M0707) CD20 (M0755), and CD68 (M0876). Mouse anti-EG2 (EG2) was from Pharmacia & Upjohn Ltd (Milton Keynes, UK).

### Quantification

In histological slides, coded to avoid observer bias, areas of epi and sub, excluding muscle and gland, were assessed using an Apple Macintosh computer and Image Version 1.55 (National Institute of Mental Health). Distinct phenotypes of inflammatory cells were counted using a Leitz Dialux 20 light microscope (Leitz Wetzlar, Wetzlar, Germany). Two to 3 bronchial biopsies for each subject were measured and counted to take account of within-subject variability. The total epithelial and subepithelial areas of 2 or 3 biopsies that were more than 0.2 mm^2^ and 1.6 mm^2^, respectively, were accepted as adequate size. The epithelial/subepithelial areas and positive cells of 2 or 3 biopsies from each bronchoscopy were summed, respectively. Then, the total counts were divided by the total area to normalize the counts as the number of cells per unit area. The data for bronchial biopsy cell counts were expressed as the number of cut cell profiles with a nucleus visible (ie, positive cells) per 0.1 mm^2^ of the epithelial area and per 1 mm^2^ of the subepithelial area. The coefficient of variation for repeat counts of cells immunopositive for subtype markers of inflammatory cells by 1 observer ranged between 5% and 6%.

### Statistical analysis

Statistical analysis was performed using StatView (SAS Institute, Inc, Cary, NC) and GraphPad Prism version 4.00 for Windows (GraphPad Software, San Diego, Calif). One-way ANOVA followed by the unpaired Student *t* test was used for the analyses of age, smoking pack-years, and lung function data between groups. In respect of cell counts in blood, sputum, and biopsies and mediators in sputum, these data were nonnormally distributed and overall differences between all groups and between 3 time points within group were assessed first using the Kruskal-Wallis test, which, if significant, was followed by Wilcoxon matched pairs test within group between baseline and infection. Differences between groups were analyzed by Mann-Whitney tests.

The coefficient of variation (=SD/mean × 100) was used to express the error of repeat cell counts in the biopsies. Spearman rank correlation was used as a test for correlations between the numbers of specific types of inflammatory cell and virus load/physiologic/clinical data/sputum inflammatory markers. A *P* value of less than .05 was accepted as indicating a significant difference. All reported *P* values are 2-sided.

## Results

### Time for peaked virus load, respiratory symptom and lung function

In subjects with COPD, individual virus load peaked on days 4 to 8 in nasal lavage, on day 5 in sputum, and on day 7 in BAL, the individual lower respiratory tract symptom and breathlessness scores peaked around day 9, and individual lowest peak expiratory flow and FEV_1_ were detected between day 5 and day 12, most of them on day 9.^E1^ The virologic and blood, sputum, or BAL inflammatory data from these subjects have been reported previously.E1, E3

### Associations between numbers of mucosal monocytes/macrophages and lymphocytes and virus load/clinical outcomes

#### Virus load

In subjects with COPD on day 7 postinfection, the numbers of sub-CD68^+^ and CD4^+^ cells were associated with peak nasal lavage virus load (*r* = 0.54 and 0.66, *P* = .027 and .007, respectively, Fig E1, *A* and *B*); sub-CD20^+^ cells correlated with peak sputum virus load (*r* = 0.57, *P* = .028, Fig E1, *C*); sub-CD8^+^ cells correlated with BAL virus load (*r* = 0.88, *P* = .013, Fig E1, *D*).

#### Clinical symptoms and lung function

In subjects with COPD only, those subjects with higher sub-CD8^+^ and CD20^+^ counts on day 7 postinfection had significantly greater peak breathlessness scores (*r* = 0.58 and 0.50, *P* = .017 and .033, respectively, Fig E2, *A* and *B*) and sub-CD68^+^ counts on day 7 correlated positively with peak lower respiratory tract symptom scores recorded between day 9 and 14 (*r* = 0.58, *P* = .021, Fig E2, *C*). In subjects with COPD, higher numbers of sub-CD8^+^, CD4^+^, and CD20^+^ cells on day 7 were significantly associated with lower prebronchodilator FEV_1_% predicted at day 9 (*r* = −0.58 to −0.73, *P* = .003-.015, Fig E2, *D-F*).
